# Pre-transplant immune factors may be associated with BK polyomavirus reactivation in kidney transplant recipients

**DOI:** 10.1371/journal.pone.0177339

**Published:** 2017-05-31

**Authors:** David DeWolfe, Jinal Gandhi, Matthew R. Mackenzie, Thomas A. Broge, Evelyn Bord, Amaara Babwah, Didier A. Mandelbrot, Martha Pavlakis, Francesca Cardarelli, Raphael Viscidi, Anil Chandraker, Chen S. Tan

**Affiliations:** 1 Division of Nephrology, Department of Medicine, Beth Israel Deaconess Medical Center and Harvard Medical School, Boston, Massachusetts, United States of America; 2 Center for Virology and Vaccine Research, Beth Israel Deaconess Medical Center and Harvard Medical School, Boston, Massachusetts, United States of America; 3 The Transplant Institute, Department of Medicine, Beth Israel Deaconess Medical Center and Harvard Medical School, Boston, Massachusetts, United States of America; 4 Department of Pediatrics, Johns Hopkins University, Baltimore, Maryland, United States of America; 5 Transplantation Research Center, Brigham and Women’s Hospital and Harvard Medical School, Boston, Massachusetts, United States of America; 6 Division of Infectious Disease Department of Medicine, Beth Israel Deaconess Medical Center and Harvard Medical School, Boston, Massachusetts, United States of America; Universidade de Sao Paulo, BRAZIL

## Abstract

BK polyomavirus (BKPyV) reactivation in kidney transplant recipients can lead to allograft damage and loss. The elements of the adaptive immune system that are permissive of reactivation and responsible for viral control remain incompletely described. We performed a prospective study evaluating BKPyV-specific T-cell response, humoral response and overall T-cell phenotype beginning pre-transplant through one year post-transplant in 28 patients at two centers. We performed an exploratory analysis of risk factors for the development of viremia and viruria as well as compared the immune response to BKPyV in these groups and those who remained BK negative. 6 patients developed viruria and 3 developed viremia. BKPyV-specific CD8^+^ T-cells increased post-transplant in viremic and viruric but not BK negative patients. BKPyV-specific CD4^+^ T-cells increased in viremic, but not viruric or BK negative patients. Anti-BKPyV IgG antibodies increased in viruric and viremic patients but remained unchanged in BK negative patients. Viremic patients had a greater proportion of CD8^+^ effector cells pre-transplant and at 12 months post-transplant. Viremic patients had fewer CD4^+^ effector memory cells at 3 months post-transplant. Exploratory analysis demonstrated lower CD4 and higher total CD8 proportions, higher anti-BKPyV antibody titers and the cause of renal failure were associated BKPyV reactivation. In conclusion, low CD4, high CD8 and increased effector CD8 cells were found pre-transplant in patients who became viremic, a phenotype associated with immune senescence. This pre-transplant T-cell senescence phenotype could potentially be used to identify patients at increased risk of BKPyV reactivation.

## Introduction

BK polyomavirus (BKPyV) is a human polyomavirus first isolated in 1971 from a kidney transplant recipient (KTR) with ureteral stenosis [1]. The virus persists latently in the renal and urinary epithelium [2]. In KTRs viral reactivation can lead to ureteral stricture or an interstitial nephritis termed BK Polyomavirus nephropathy (BKN)[3, 4]. BKPyV reactivation in blood (viremia) is detected in up to 50% of KTRs with BKN occurring in approximately 10% [5, 6]. BKN is associated with high rates of graft loss [7–11], and viremia is associated with acute rejection, declining allograft function [11] and the development of *de novo* donor specific antibodies [12].

Currently, it is recommended that all KTRs be screened for BKPyV by PCR of urine or blood post-transplant [8, 13]. The only treatment known to be efficacious is reduction in immune suppression (IS)[14], which carries with it the risk of acute rejection [15]. Previous studies have demonstrated low or negative anti-BKPyV antibodies [16, 17] and low or absent BKPyV-specific T-cells prior to transplant [8, 18, 19] are risk factors for BKPyV reactivation. The development of BKPyV-specific T-cells without IS reduction has been associated with self-limited viremia, and failure to develop BKPyV-specific cellular response is associated with prolonged viremia and BKN [20, 21]. Rising anti-BKPyV IgG and IgM antibody titers are associated with viral reactivation and correlate with severity of disease [22–25].

Although previous studies have evaluated the BKPyV-specific T-cell response, detailed longitudinal understanding of such response in context of clinical characteristics and outcomes is lacking. Furthermore, no studies have attempted to evaluate pre-transplant T-cell phenotypes in order to establish whether specific profiles may alter reactivation risk. We hypothesized that risk of developing BKV-associated diseases post-transplant may in part be determined by specific immune factors pre-transplant. In this exploratory study, we prospectively followed 28 patients who underwent renal transplantation at two local institutions. We assessed the presence of BKPyV-specific humoral and cellular immune response before transplant and for one year post-transplant to identify early BKPyV-specific immune alterations to identify those who were protected against BKPyV viremia or reactivation limited to the urine (viruria). Additionally, we performed an immuno-phenotype analysis of T-cells to identify pre-transplant phenotypic alterations which may be permissive of or protective against viral reactivation.

## Methods

### Subjects and sample collection

This prospective observational cohort study was approved by the internal review boards of Beth Israel Deaconess Medical Center and the Brigham and Women’s Hospital. Patients were enrolled at the transplant clinics of both institutions from September 2012 to October 2014. Urine and peripheral blood samples were collected before kidney transplantation and 1, 3, 6 and 12 months post-transplant. Plasma and peripheral blood mononuclear cells (PBMC) were isolated and aliquots of PBMC, plasma and urine were stored at -80°C. Demographic and clinical information, including BKPyV urine and serum PCR screening values, were collected from the medical record.

### Intracellular cytokine staining (ICS)

PBMC were separated by Ficoll-Paque gradient centrifugation, washed, and resuspended in RPMI-1640 with 12% fetal calf serum media to a concentration of 3.5 × 10^6^ cells/ml. PBMC (7 × 10^6^) were plated without peptides to serve as the negative control, or stimulated with BKPyV VP1 peptide pool of 15mers overlapping by 11, covering the entire VP1 capsid (JPT, Germany), at a final concentration of 2μg/mL. Cells were cultured for 10–14 days; after the first 96 hours, the medium was supplemented with 25 U/ml interleukin-2 (IL-2). After this time, 1x10^6^ lymphocytes were washed twice to remove IL-2, and plated in RPMI 1640 with 12%FBS (negative control), with BKPyV peptide pool (2μg/mL), or PMA (1μg/μL) and Ionomycin (5μg/μL) as positive control, at 37°C for 6 hours. After the first hour, all cells received monensin (GolgiStop; BD Biosciences).

Cells were stained with fluorescently-conjugated antibodies specific for human CD4 (clone L200) and CD8 (clone SK1). They were then permeabilized, stained for IFN-γ (clone B27) and CD3 (clone SP34.2), and finally fixed. All antibodies were obtained from BD Biosciences. Data were collected using a LSRII flow cytometer (BD Biosciences) and analyzed with FlowJo software (Treestar Inc.). ICS results were considered positive if the percentage of IFN- γ producing CD4^+^ or CD8^+^ T cells was equal to or greater than two times the baseline value of IFN-γ. ICS results are reported as the difference between the baseline and experimental IFN-γ values.

### Humoral response to BKPyV

BKPyV pseudovirion-coated ELISA was used to quantify anti-BKPyV IgG against BKPyV serotype I as has been previously described[26]. Results were reported as mean values of duplicate reactions. The serum dilution in assays for anti-BKPyV IgG was 1:100. The cut-off for seropositivity was an OD value >0.20.

### T lymphocyte phenotyping

A 100μl aliquot of whole blood was stained with fluorescently-conjugated antibodies specific for human CD3 (clone SP34.2 BD Biosciences), CD4 (clone L200 BD Biosciences), CD8 (clone RPA-T8 BioLegend), CD45RO (clone UCHL1 Beckman Coulter), CD27 (clone M-T271 BD Biosciences), CD38 (clone HB7 BD Biosciences), HLA-DR (clone L243 BD Biosciences), and PD-1 (clone EH12.2H7 BioLegend). Samples were run through TQ-Prep machine (Beckman Coulter) to lyse red blood cells, washed in D-PBS, and fixed in 1.5% formaldehyde. Data were collected by running the samples through multicolor flow cytometry on a LSRII flow cytometer (BD Biosciences), and analyzed with FlowJo software (Treestar Inc.) The gating strategy was as follows:T-cells were identified by forward vs side scatter, excluded of doublets, and further gated on CD3^+^ cells, which were then subdivided into CD3^+^/CD4^+^ and CD3^+^/CD8^+^ populations. Those, in turn, were subdivided by receptor status into naïve cells (CD45RO^-^/CD27^+^), effector cells (CD45RO^-^/CD27^-^), effector memory cells (CD45RO^+^/CD27^-^) and central memory cells (CD45RO^+^/CD27^+^). Each subset was evaluated for expression of activation markers CD38 and HLA-DR, and for exhaustion marker PD-1.

### BKPyV DNA Detection

DNA extraction was performed with the QIAamp MinElute Virus Spin Kit (Qiagen, CA) following kit protocol. BKPyV DNA was quantified by Quantitative PCR (qPCR) using 7300 Real Time PCR System (Applied Biosystems, CA). The primer pair 5′-AGTGGATGGGCAGCCTATGTA-3′ (nt 2511–2531) and 5′-TCATATCTGGGTCCCCTGGA-3′ (nt2586–2605), and probe 6FAM-AGGTAGAAGAGGTTAGGGTGTTTGATGGCACA-TAMRA (nt 2546–2578) (Applied Biosystems, CA), located in the VP1 gene, were used for qPCR detection, as previously described [27], with a C to G modification of nucleotide 2569. For each serum or urine sample, the extraction volume was 200μl and the elution volume was 150μl. Each qPCR reaction was run in triplicate and all results were expressed in copies/ml.

### Data analysis

Categorical independent variables were compared using Fisher’s exact test and data are presented as number (N) and percent (%) of population. Continuous and ordinal independent variables were compared using the Kruskal-Wallis test and data are presented as means and standard deviations. Longitudinal variables were analyzed using linear or logistic generalized estimating equations (GEE)[28] in order to control for intra-individual correlations introduced by repeated measures. To identify variables associated with viremia and viruria, an exploratory univariable logistic regression screen was utilized with an inclusion P value cut off of ≤ 0.15[29] for multivariable analysis. A stepwise selection process starting with the candidate variable with the lowest P value was undertaken to identify variables for multivariable analysis. Due to a low event number, a multivariable analysis for viremia as the outcome was not performed, rather a combined outcome of viruria and viremia together was analyzed. Variables were added in a step-wise fashion based upon their P value and retained in the model if their P value was ≤ 0.10. This process proceeded until all variables had a P value ≤0.10 or were identified as significant confounding variables. Logistic regression data are presented as an odds ratio with 95% CI and P value. All P values presented are two-tailed P values with P < 0.05 considered statistically significant. Statistical analysis performed on Stata version 14 statistical software, StataCorp LP, College Station, Tx. Graphics performed on GraphPad Prism Version 5.0 software, GraphPad Software, La Jolla CA.

## Results

### Patient characteristics and outcomes

29 patients were enrolled and 28 underwent transplantation. One transplant was canceled due to donor complications. Patients were retrospectively stratified into groups with no viral reactivation (BK Negative), viruria only and viremia. No significant differences were found in recipient, donor or transplant characteristics between the 3 groups (Table 1). 7/28 (25%) patients had BKPyV reactivation in either urine or blood by standard of care clinical screening. Two additional patients were found to be viruric by research lab qPCR, making 9 (32%) total reactivations. 6 (21%) developed viruria only and 3 (11%) developed viremia (Table 2). 1/29 (4%) patient was found to have viruria prior to transplant. 3 patients developed viruria within the first month post-transplant and all reactivations occurred within 6 months. Viremia developed between 3 and 6 months in all 3 cases with peak viral loads of 27,022 copies/ml, 2,111 copies/ml and 19,031 copies/ml. There were no cases of sustained viremia or BKN in the study period. All 3 viremic patients were treated with IS reduction and 2/3 (67%) were treated with Leflunomide. 1/6 (17%) viruric patient was treated with IS reduction. Viremia patients had a trend to higher serum creatinine at 12 months post-transplant at 1.60 (±0.26) mg/dL compared to viruria patients at 1.22 (±0.41) mg/dL and BK negative at 1.31 (±0.30) mg/dL (P = 0.20), but this did not attain statistical significance. There were 4 (14%) patients who developed acute rejection, none of whom developed BKPyV reactivation.

**Table 1:** Study subject characteristics.

	BK Viremia	BK Viruria	BK Negative	P Value
**Number of Patients**	3	6	19	
**Recipient Age (Years)**	45.71 (± 19.40)	54.68 (± 14.53)	51.3 (± 11.73)	P = 0.72
**Recipient Gender**				
Male	3	4	13	
Female	0	2	6	P = 0.68
**Recipient Race**				
Caucasian	2	5	18	
African American	1	0	1	
Asian	0	1	0	P = 0.18
**Cause of Renal Disease**				
Diabetes	2	3	7	
Primary GN	1	3	1	
ADPKD	0	0	4	
Obstruction/Pyelonephritis	0	0	3	
Other	0	0	4	P = 0.21
**On RRT at time of transplant**				
Yes	2	1	4	
No	1	5	11	P = 0.29
**Recipient CMV Serostatus**				
Positive	2	2	3	
Negative	1	4	16	P = 0.11
**Induction Immune Suppression**				
rATG	2	5	15	
Basiliximab	1	1	2	P = 0.73
**Corticosteroid Withdrawal**				
No	2	0	4	
Yes	1	6	15	P = 0.10
**Donor Age (Years)**	58.20 (± 3.38)	43.54 (± 14.40)	45.06 (± 11.42)	P = 0.14
**Type of Transplant**				
LRRT	1	4	7	
LURT	2	2	11	
DDRT	0	0	1	P = 0.66
**Donor Gender**				
Male	0	2	5	
Female	3	4	14	P = 0.82
**Donor Race**				
Caucasian	2	5	18	
African American	1	0	0	
Asian	0	1	0	P = 0.06
**Warm Ischemia Time (min)**	34.00 (± 9.86)	41.50 (± 15.80)	50.97 (± 30.43)	P = 0.56
**Cold Ischemia Time (min)**	168.67 (± 243.66)	30.50 (± 12.22)	65.36 (±91.89)	P = 0.26

Categorical variables were compared using Fisher’s exact test and data are presented as number (N). Continuous and ordinal independent variables were compared using the Kruskal-Wallis test and data are presented as means and standard deviations. GN = glomerulonephritis; ADPKD = autosomal dominant polycystic kidney disease; RRT = renal replacement therapy; rATG = rabbit anti-thymocyte globulin; LRRT = living related renal transplant; LURT = living unrelated renal transplant; DDRT = deceased donor renal transplant.

**Table 2:** Prevalence of BKPyV detected in the plasma or urine.

Time	Plasma	Urine
**Pre-Transplant**	0/29 (0%)	1/29^Ŧ^ (3%)
**1 month**	0/28 (0%)	3/28 (10.7%)
**3 months**	2/28 (7.1%)	4/28 (14.3%)
**6 months**	2/28 (7.1%)	5/28 (17.9%)
**12 months**	0/27 (0%)	2/27* (7.4%)

^Ŧ^: 1 patient’s transplant was canceled due to donor complications.

*: 1 patient received a pancreas after kidney transplant while viruric, he remained viruric only at 1 year, but is not counted in our study after receiving the pancreas.

### BKPyV-specific T cell responses

Pre-transplant 20/21 (95%) tested patients had detectable CD4^+^ BKPyV-specific T-cells, including 12/12 (100%) BK negative and 6/6 (100%) viruric patients, and 2/3 (67%) viremic patients. All viruric and viremic patients developed CD4^+^ BKPyV-specific T-cells by 3 months and maintained them through 12 months. Two BK negative patients transiently lost detectable BKPyV-specific CD4^+^ T-cells post-transplant. 16/21 (76%) patients had CD8^+^ BKPyV-specific T-cells at pre-transplant. All viremic patients and 5/6 (83%) viruric patients had BKPyV-specific CD8^+^ T-cells, but only 8/12 (67%) BK negative patients did. At 1 month post-transplant, only 1/3 (33%) viremic patients had detectable CD8^+^ BKPyV-specific T-cells, while the prevalence did not change in viruric or BK negative patients. By 3 months, all patients with viral reactivations had CD8^+^ BKPyV-specific T-cells and maintained them through 12 months. The prevalence of CD8^+^ BKPyV-specific T-cells in BK negative patients remained less throughout the followup period at under 50%, though this did not reach statistical significance.

**Fig 1:**
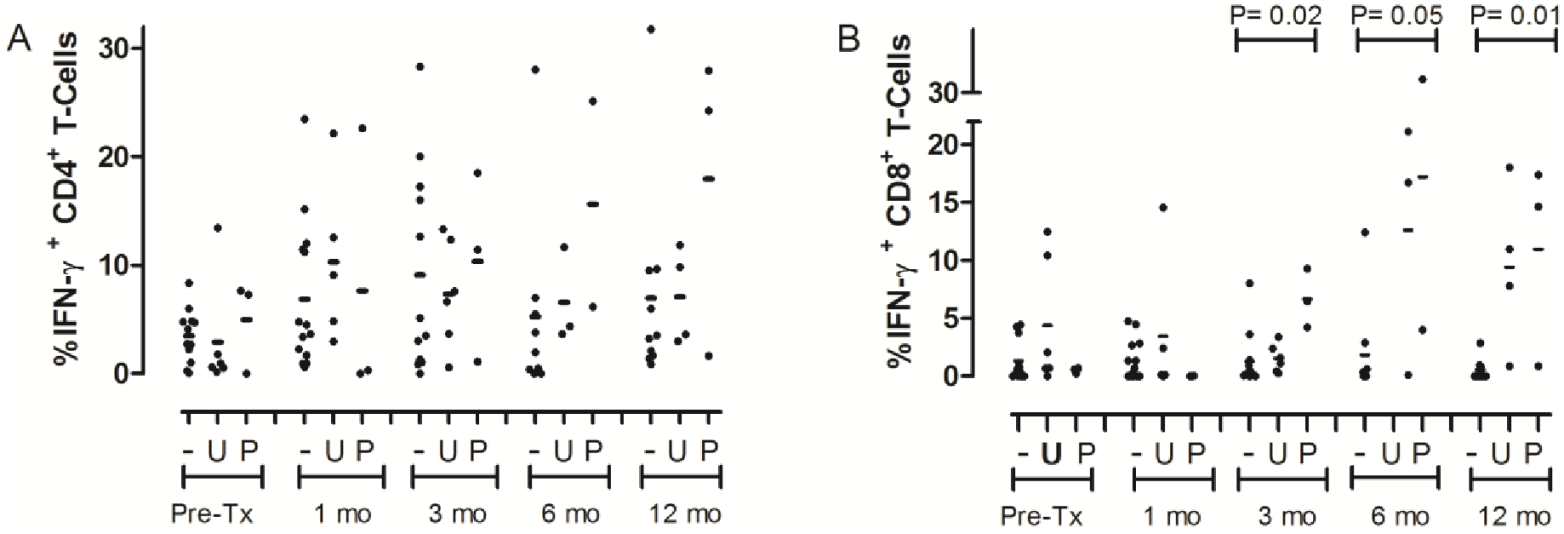
BKPyV specific (A) CD4^+^ and (B) CD8^+^ T-cells. Line represents mean value. “-” = BK Negative, “U” = viruria, “P” = viremia, “mo” = months post-transplant. “Pre-Tx” = Pre-transplant. P values were calculated by Kruskall-Wallis comparing all 3 groups at each time point. **A**: No significant difference at any time point. **B**: 3 month mean %IFN-γ producing CD8^+^ T-Cells for BK negative 1.29 (±2.36), viruria 1.54 (±1.21), viremia 6.67 ±0.54), P = 0.02; 6 months mean for BK negative 1.81 (±4.10), viruria 12.65 (±11.10) and viremia 17.22 (±18.68), P = 0.05; 12 months mean for BK negative 0.53 (±0.91), viruria 9.44 (±7.13), viremia 10.97 (±8.85), P = 0.01.

The change in percent T-cells which were BKPyV-specific over time was evaluated utilizing linear GEE regression (Table 3). BKPyV-specific CD4^+^ T-cells did not change in BK Negative patients (0.10%/month; P = 0.64) or viruric patients (0.11%/month; P = 0.63) but significantly increased in viremic patients (1.03%/month; P = 0.04). BKPyV-specific CD8^+^ T-cells increased with time both in viremic patients (1.02%/month; P = 0.02), and viruric patients (0.51%/month; P = 0.05). There was no significant change in BK negative patients (-0.03%/month; P = 0.62). Utilizing Kruskal-Wallis to compare the percent CD4^+^ BKPyV-specific T-cells between the three viral reactivation groups at each experimental time point, there was a non-significant trend for higher counts in viremic patients (Fig 1A). The percent CD8^+^ BKPyV-specific T-cells were higher in viremic (6.67±0.54) than viruric (1.54 ±1.21) and BK negative (1.29 ±2.36) patients at 3 months (P = 0.02) post-transplant and, higher in viremic and viruric patients than BK negative at 6 (BK negative 1.81 ±4.10, viruric 12.65 ±11.10 and viremic17.22 ±18.68; P = 0.05) and 12 (BK negative 0.53 ±0.91, viruric 9.44 ±7.13, viremic 10.97 ±8.85; P = 0.01) months post-transplant (Fig 1B).

**Table 3:** GEE Linear regression for change in variable per month post-transplant.

BKV Reactivation Status	Coefficient	P Value	Coefficient	P Value	Coefficient	P Value
	IFN-γ+ CD4 (% per month)		IFN-γ+ CD8 (% per month)		Anti BKV IgG (OD/month)	
**BK Negative**	0.10	0.64	-0.03	0.62	0.01	0.17
**Viruria**	0.11	0.63	0.51	0.05	0.08	<0.01
**Viremia**	1.03	0.04	1.02	0.02	0.07	<0.01

OD = optical density, IFN-γ+ = interferon gamma positive

### T cell immuno-phenotype

**Fig 2:**
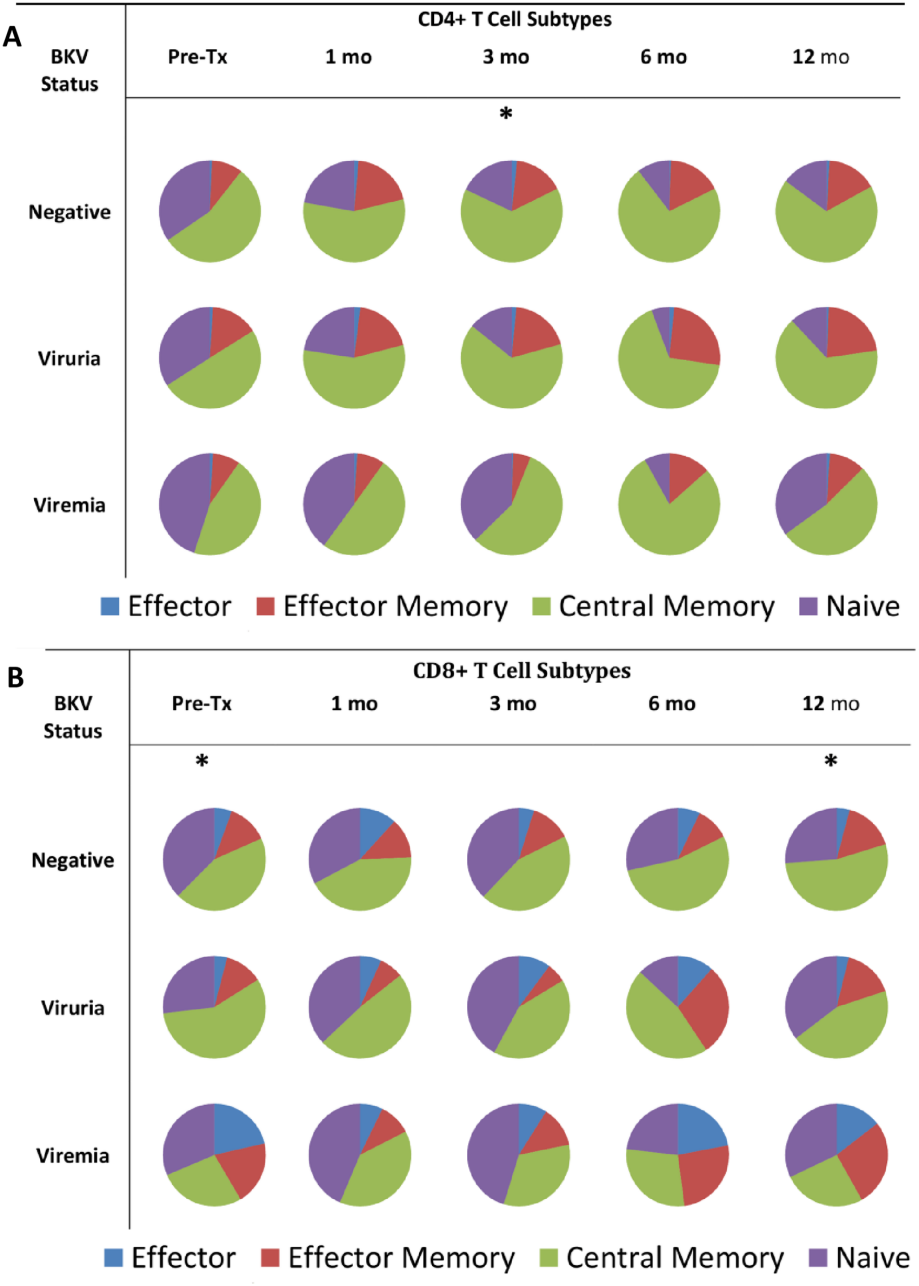
Phenotypic composition of T lymphocytes in the BK negative, viruria, and viremia groups. **2A: CD4+ T-Cell Phenotypes** *P < 0.05. “Pre-Tx” = Pre-transplant. “mo” = Months post-transplant. P values were calculated by Kruskall-Wallis comparing each T-cell subgroup at each time point. Effector Cells are CD45RO-/CD27-; Effector Memory Cells are CD45RO+/CD27-; Central Memory Cells are CD45RO+/CD27+; Naïve Cells are CD45RO-/CD27+. Expressed are means as a percent of total CD4+ T-Cells, may not add to 100% due to rounding. *Effector memory cells at 3 months in BK negative 15.93% (±11.29), viruria 20.00% (±12.00) and viremia 5.59% (±2.32) P = 0.05. **2B: CD8+ T-Cell Phenotypes** *P < 0.05. “Pre-Tx” = Pre-transplant. “mo” = Months post-transplant. P values were calculated by Kruskall-Wallis comparing each T-cell subgroup at each time point. Effector Cells are CD45RO-/CD27-; Effector Memory Cells are CD45RO+/CD27-; Central Memory Cells are CD45RO+/CD27+; Naïve Cells are CD45RO-/CD27+. Expressed are means as a percent of total CD8+ T-Cells, may not add to 100% due to rounding. *Baseline percent effector cells for viremia is 21.41% (±13.11), viruria 4.04% (±4.51) and BK negative is 5.49% (±4.76), P = 0.04. 12 month percent effector cells for viremia is 14.49% (±3.76), viruria 3.86% (±1.64), and BK negative 4.10% (±4.19), P = 0.01.

CD4 and CD8 circulating T-cells were classified into their functional subtypes of central memory, effector memory, effector and naïve cells as described above (Fig 2A and 2B, S1 and S2 Tables). Viremic patients demonstrated a greater proportion of CD8^+^ T-cells with an effector phenotype pre-transplant (21.41% ±13.11) compared to viruric (4.04% ± 4.51) and BK negative (5.49% ±4.76)(P = 0.04). This was also true at 12 months (viremic 14.49% ±3.76, viruric 3.86% ±1.65, BK negative 4.10% ±4.19, P = 0.01) (Fig 2B). No significant differences were observed among the other CD8 subsets. Viremic patients demonstrated fewer CD4^+^ T-cells with an effector memory subtype at 3 months post-transplant (5.59% ±2.32) compared to BK negative (15.93% ±11.29) and viruric (20.00% ±12.00) (P = 0.05) (Fig 2A). No other significant differences were observed among the other CD4 subsets.

Each of the subtypes of both CD4^+^ and CD8^+^ T-cells were analyzed for the expression of activation markers CD38 and HLA-DR as well as the exhaustion marker PD-1. At one month post-transplant, viremic patients were found to have fewer CD4^+^ central memory cells displaying the activation markers CD38 and HLA-DR. Fewer CD4^+^ central memory cells in viremic patients expressed CD38 (38.04% ±15.90) compared to BK negative (57.10% ±12.01) and viruric (60.07% ±14.67) (P = 0.04). Similarly, fewer CD4^+^ central memory cells in viremic patients expressed HLA-DR (22.65% ±12.08), compared to viruric (39.33 ±7.42) and BK negative (33.84±7.77) (P = 0.03). There were no other significant differences among activation markers found in any other subset. There were no significant differences among any subset expressing exhaustion marker PD-1.

### Anti-BKPyV IgG response

The humoral response to BK virus was assed via ELISA for IgG antibodies against the most prevalent BK virus serotype in North America, BKPyV I[30]. Anti-BKPyV IgG was present pre-transplant in 13/15 (87%) BK negative patients, 3/3 (100%) viremic and 5/6 (83%) viruric patients. At 1 month post-transplant, 3/3 (100%) viremic, 5/5 (100%) viruric and 14/15 (93%) BK negative patients had anti-BKPyV IgG.

**Fig 3:**
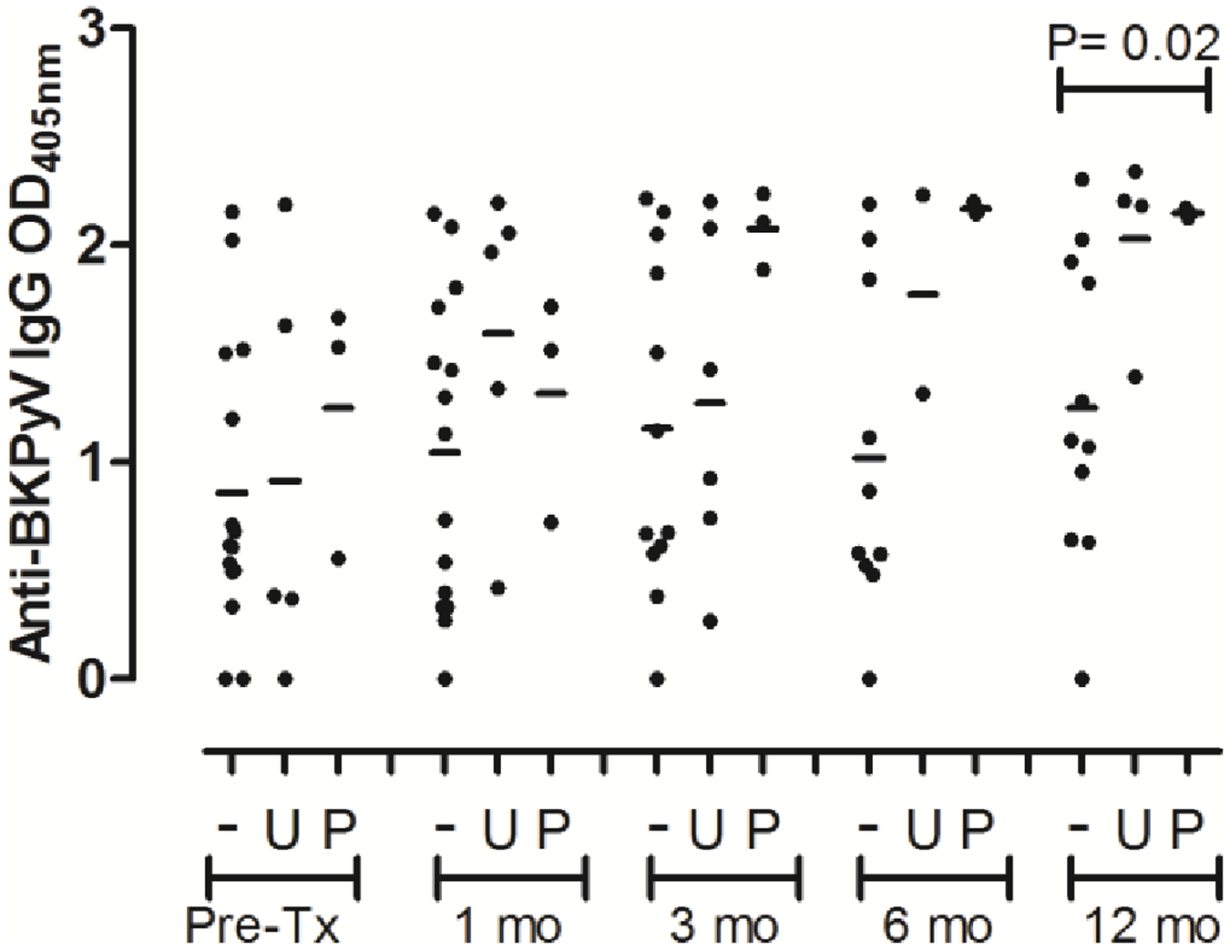
Anti-BKPyV IgG. Line represents mean value. “OD” = Optical Density, “-” = BK Negative, “U” = viruria, “P” = viremia, “mo” = months post-transplant. “Pre-Tx” = Pre-transplant. P values were calculated by Kruskall-Wallis comparing all 3 groups at each time point. 12 month mean for BK negative 1.25 (± 0.70), viruria 2.03 (± 0.43), viremia 2.15(± 0.02), P = 0.02.

Linear GEE regression demonstrated anti-BKPyV IgG increased with time for viruric patients (0.08 units/month; P<0.01) and viremic patients (0.07 units/month; P <0.01), but did not change in BK negative patients (0.01 units/month; P = 0.17) (Table 3). Utilizing the Kruskal-Wallis test, while the mean IgG against BKPyV was higher among viruric and viremic than BK negative patients at 3, 6 and 12 months, this did not reach significance until month 12 (BK negative 1.25 ± 0.70, viruria 2.03 ± 0.43, viremia 2.15 ± 0.02; P = 0.02) (Fig 3).

### Factors associated with BKPyV reactivation

In an exploratory analysis, the clinical and experimental variables were screened utilizing GEE logistic regression to evaluate their association with BKPyV reactivation, a combined endpoint of viremia and viruria (Table 4). Of the baseline characteristics, the underlying reason for renal failure was significantly associated with viral reactivation, with 4/5 (80%) patients with glomerulonephritis (GN) and 5/12 (42%) patients with diabetes mellitus accounting for all viral reactivations (P = 0.03). Of the experimental variables, several were significant predictors in univariable analysis, including IgG titers against BKPyV, CD4^+^ and CD8^+^ BKPyV-specific T-cells and CD4 and CD8 proportions. The multivariable analysis identified the cause of renal failure (OR 0.44; 95% CI 0.19–0.99, P< 0.05), anti-BKPyV IgG (OR 4.01; 95% CI 1.54–10.46, P<0.01), and percent of T-cells which are CD8^+^ (OR 1.04; 95% CI 1.00–1.07, P = 0.05) and CD4^+^ (OR 0.95; 95% CI 0.92–0.98, P<0.01) as associated with viral reactivation. Immune suppression and trough levels were not significant predictors.

**Table 4:** GEE logistic regression for BKPyV reactivation.

Variable	OR	95% CI	P	OR	95% CI	P
	Univariable Analysis			Multivariable Analysis		
**Month**	1.08	1.02–1.13	<0.01			
**MMF Dose**	0.99	0.99–0.99	0.01			
**Recipient CMV Status**	3.65	0.77–17.28	0.10			
**Recipient Race**	0.31	0.07–1.48	0.14			
**Donor Race**	0.19	0.02–1.53	0.12			
**Cause of Renal Failure**	0.48	0.24–0.95	0.03	0.44	0.19–0.99	<0.05
**Anti BKPyV IgG**	3.98	1.77–8.93	<0.01	4.01	1.54–10.46	<0.01
**Per % CD4 IFN-γ+ T-Cells**	1.05	1.01–1.10	0.03			
**Per % CD8 IFN-γ+ T-Cells**	1.08	1.01–1.15	0.03			
**Per % CD3+ T-Cells**	0.98	0.97–1.00	0.03			
**Per % CD8+ T-Cells**	1.04	1.01–1.06	<0.01	1.04	1.00–1.07	<0.05
**Per % CD4+ T-Cells**	0.94	0.91–0.96	<0.01	0.95	0.92–0.98	<0.01

OR = Odds Ratio. CI = Confidence Interval. Percent CD3+ T-Cells, CD4+ T-Cells and CD8+ T-Cells are the percent of peripheral blood mononuclear cells (PBMC) which are CD3+ (T-Cells), CD4+ or CD8+ respectively. Percent CD4 IFN-γ+ and Percent CD8+ IFN-γ+ T-Cells are the percent of cultured PBMC which secrete interferon gamma in response to BKPyV antigen stimulation. Cause of Renal Failure, see Table 1. MMF = mycophenolate mofetil.

### Factors associated with BK viremia

A univariable screen was repeated to identify variables associated with BK viremia alone. In the univariable screen, anti-BKPyV IgG (OR 3.16; 95% CI 1.05–9.48, P = 0.04), and CD4 (OR 0.95; 95% CI 0.90–0.99, P = 0.02) and CD8 (OR 1.03; 95% CI 1.00–1.06, P = 0.05) proportions were significantly associated with development of viremia (Table 5). No clinical characteristics or immune suppression medications or troughs were significant predictors. A multivariable model was not attempted due to the low number of viremia patients.

**Table 5:** Univariable GEE logistic regression for BKPyV viremia.

Variable	OR	95% CI	P
	Univariable Analysis		
**Anti BKPyV IgG**	3.16	1.05–9.48	0.04
**Per % CD8+ T-Cells**	1.03	1.00–1.06	0.05
**Per % CD4+ T-Cells**	0.95	0.90–0.99	0.02

OR = Odds Ratio. CI = Confidence Interval. Percent CD4+ T-Cells and Percent CD8+ T-Cells are the percent of peripheral blood mononuclear cells (PBMC) which are CD3+ (T-Cells) and CD4+ or CD8+ respectively.

## Discussion

BKN is an important and preventable cause of allograft dysfunction and loss. We have performed a longitudinal analysis of the adaptive immune response to BKPyV reactivation, including the timing and magnitude of the BKPyV-specific T-cell response in both CD4^+^ and CD8^+^ cells and the humoral immune response. Additionally, we quantified T-cell phenotypes to attempt to identify a profile which would be permissive of viral reactivation. Lastly, we evaluated these experimental factors alongside clinical data to fully assess risk of viral reactivation.

Previous work has demonstrated that a robust CD4^+^ T-cell response to viral capsid proteins is associated with previously resolved BKPyV reactivation [24, 31–33], and an early post-transplant CD8^+^ T-cell response[34], specifically polyfunctional CD8^+^ T-cells[35] is associated with viral control. Our data demonstrate that the presence of CD4^+^ and CD8^+^ BKPyV-specific T-cells is common in the pre-transplant population, and that the prevalence of BKPyV-specific T-cells drops immediately after transplant. Our data echoes prior studies which suggest the absence of CD4^+^, CD8^+^, or both BKPyV-specific T-cells pre- and early post-transplant may be a risk factor for viral reactivation[19]. We have shown an increase in both CD4^+^ and CD8^+^ BKPyV-specific T-cells in viremic patients and that this is associated with clearance of viremia in all cases. Our data also show a significant, though less substantial, CD8^+^ BKPyV-specific T-cell response in those with viruria, also associated with viral control. Our findings suggest a role for monitoring the emergence of BKPyV-specific T-cells in those with BK reactivation identified by screening. This would serve as a guide to identifying those with an adequate response and likely, therefore, to have self-limited reactivation and in whom IS reduction is unnecessary. Additionally, tracking BKPyV-specific T-cells could be used to guide the magnitude and monitor the effectiveness of step-wise IS reduction to prevent BKPyV progression and prevent excess risk of allograft rejection.

Our data adds to prior clinical observations indicating that having experienced a humoral response does not give full protection from post-transplant viral replication [24, 36]. It also confirms the course of BKPyV-specific antibody response follows the level and duration of BKPyV replication [22, 24, 25, 37]. By the first month post-transplant, all patients with viral reactivation had positive anti-BKVPyV antibodies, and we demonstrated those titers increased in viremic and viruric patients with time, but remained unchanged in BK negative patients.

We further evaluated circulating T lymphocytes by phenotype, which revealed several important distinctions between the groups. The proportion of T-cells that are CD4^+^ was lower and CD8^+^ T-cells were higher in viremic patients than viruric or BK negative patients before transplant. Furthermore, a significantly greater proportion of CD8^+^ T-cells had a terminally differentiated effector phenotype prior to transplant in viremic patients, a phenotype they regained several months after transplant. Studies have identified an increased fraction of terminally differentiated CD8^+^ T-cells along with low proportions of CD4^+^ and high proportions of CD8^+^ T-cells as a marker of both age [38, 39] and uremia associated [40, 41] immune senescence. Our results suggest that prior to ever receiving an immune suppressive regimen, patients who later become viremic demonstrate a T-cell profile associated with immune senescence.

Our analysis of clinical and experimental factors associated with BKPyV reactivation and viremia identified markers of increased immune suppression, such as the presence of diabetes mellitus or GN, conditions associated with immune dysfunction or immune dysregulation [42–45], as the cause of renal failure, and of immune senescence, such as lower proportions of CD4 T-cells and higher proportions of CD8 T-cells. They also include potential markers of increased BKPyV burden, such as increased anti-BKPyV IgG titers.

Our exploratory analyses demonstrate lower CD4 and higher CD8 proportions were risk factors for viremia and viruria and was also seen pre-transplant. CD4 T-cells are typically two-fold higher than CD8 T-cells, and a drop in CD4/CD8 ratio has been associated with immune senescence and mortality in elderly individuals [38]. This same profile has been described in CKD patients irrespective of whether they are yet on RRT or RRT modality, and does not improve post-transplant [40, 41, 46–48]. Immunologic aging and senescence are also associated with reduced naïve cells and expansion of terminally differentiated effector CD8^+^ T-cells [48, 49], a feature which was also present in viremic patients in our analysis. This leads to estimates that the adaptive immune system of ESRD patients is equivalent to healthy individuals 20–30 years older [46, 50]. Furthermore, these phenotypic changes seem to be driven or exacerbated by persistent CMV infection [38, 49]. The combination of low CD4/CD8 ratio and CMV seropositivity has been identified as an “immunological risk profile” and recently shown to predict severe bacterial infections and opportunistic infections after kidney transplant [51]. It has also been associated with a decreased risk of acute rejection [52]. Our study demonstrates for the first time in the literature that patients with features of an immune senescence phenotype can be identified pre-transplant and that these persons may be at increased risk of BKPyV reactivation. Additional clinical information, such as causes of renal failure associated with immune suppression may further inform the risk profile.

Limitations of our study include a small number of patients, reducing the power of the study to statistically identify all apparent differences. The small event number is particularly important for our exploratory logistic analysis of risk factors for BKPyV reactivation and viremia. The screening of large numbers of clinical and experimental variables for these analyses increases the risk of type 1 error, and these analyses should be viewed as hypothesis generating. Our patients were from two centers, utilizing mostly rATG and an early corticosteroid withdrawal protocol, almost all from living donors and a high percentage were preemptive transplants, potentially limiting generalizability. The frequency of BKPyV-specific T cells is low, requiring ex-vivo stimulation to study, limiting the clinical applicability of this method, though it is a common method used for study and is thought to represent the in vivo conditions[53]. Our longitudinal data were collected prospectively and reflect “real world” conditions of screening and response to intervention; they also incorporate both experimental and clinical data. Our study serves as a hypothesis generating pilot study, and results need to be replicated in larger studies. Further research on larger numbers of patients, in patients with non-living donors and in BKN patients is needed.

## Supporting information

S1 Table CD4 T-cell phenotype. Means and standard deviation of CD4^+^ T-cell phenotypes as described in Fig 2A.
(DOCX)

S2 Table CD8 T-cell phenotype. Means and standard deviation of CD8^+^ T-cell phenotypes as described in Fig 2B.
(DOCX)

S1 Dataset All obtained data. All collected and de-identified data used for analysis.
(DTA)
