# Supplementary material for: Pre-transplant immune factors may be associated with BK polyomavirus reactivation in kidney transplant recipients
Source: PLoS One. 2017 May 31;12(5):e0177339. doi: 10.1371/journal.pone.0177339 (PMC5451008; doi:10.1371/journal.pone.0177339)
Supplement: S1 Table — Means and standard deviation of CD4+ T-cell phenotypes as described in Fig 2A. (DOCX) [file pone.0177339.s001.docx]

**Table S1: CD4 T-cell Phenotype**

|  | BK Negative | Viruria | Viremia | P |
| --- | --- | --- | --- | --- |
| Baseline |  |  |  |  |
| Total CD4+ | 70.72±9.40 | 63.07±10.55 | 58.81±7.00 | 0.10 |
| Naïve | 34.44±20.26 | 33.48±20.83 | 45.02±26.73 | 0.72 |
| Central Memory | 54.68±16.38 | 50.26±10.27 | 45.21±22.06 | 0.75 |
| Effector Memory | 9.95±6.36 | 15.23±15.38 | 8.71±4.48 | 0.90 |
| Effector | 0.65±0.71 | 1.04±0.99 | 1.05±0.97 | 0.37 |
| 1 Month |  |  |  |  |
| Total CD4+ | 47.73±16.22 | 38.90±20.14 | 44.58±15.16 | 0.32 |
| Naïve | 20.74±17.43 | 22.53±17.05 | 39.92±29.51 | 0.39 |
| Central Memory | 57.76±14.56 | 56.55±14.21 | 50.27±25.86 | 0.97 |
| Effector Memory | 20.05±12.59 | 18.92±13.09 | 8.84±3.55 | 0.17 |
| Effector | 1.45±1.88 | 2.00±1.85 | 0.97±0.66 | 0.71 |
| 3 Months |  |  |  |  |
| Total CD4+ | 45.06±9.14 | 43.38±18.02 | 45.66±14.53 | 0.75 |
| Naïve | 17.94±16.15 | 14.79±10.01 | 37.35±38.11 | 0.66 |
| Central Memory | 64.37±12.99 | 63.72±15.35 | 56.59±37.37 | 0.95 |
| Effector Memory | 15.93±11.29 | 19.99±11.99 | 5.59±2.31 | 0.05* |
| Effector | 1.72±3.19 | 1.49±1.36 | 0.47±0.62 | 0.38 |
| 6 Months |  |  |  |  |
| Total CD4+ | 41.86±8.91 | 48.39±24.84 | 27.74±2.02 | 0.32 |
| Naïve | 10.38±.82 | 5.71±1.18 | 7.99±6.32 | 0.83 |
| Central Memory | 71.98±9.41 | 66.93±19.68 | 78.62±1.78 | 0.54 |
| Effector Memory | 17.08±7.83 | 25.82±18.35 | 13.22±4.44 | 0.45 |
| Effector | 0.55±0.45 | 1.54±2.15 | 0.18±0.08 | 0.25 |
| 12 Months |  |  |  |  |
| Total CD4+ | 54.68±11.10 | 54.22±14.35 | 40.23±22.11 | 0.53 |
| Naïve | 14.84±15.57 | 11.95±10.33 | 34.96±37.35 | 0.35 |
| Central Memory | 68.24±11.48 | 65.38±15.36 | 52.54±36.62 | 0.93 |
| Effector Memory | 15.93±7.67 | 21.92±20.72 | 11.39±2.16 | 0.58 |
| Effector | 0.99±1.59 | 0.75±1.25 | 1.11±1.44 | 0.70 |
