# Supplementary material for: Pre-transplant immune factors may be associated with BK polyomavirus reactivation in kidney transplant recipients
Source: PLoS One. 2017 May 31;12(5):e0177339. doi: 10.1371/journal.pone.0177339 (PMC5451008; doi:10.1371/journal.pone.0177339)
Supplement: S2 Table — Means and standard deviation of CD8+ T-cell phenotypes as described in Fig 2B. (DOCX) [file pone.0177339.s002.docx]

**Table S2: CD8 T-cell Phenotype**

|  | BK Negative | Viruria | Viremia | P |
| --- | --- | --- | --- | --- |
| Baseline |  |  |  |  |
| Total CD8+ | 31.57±19.36 | 28.42±4.16 | 37.99±7.43 | 0.20 |
| Naïve | 37.40±27.23 | 26.84±31.07 | 31.37±23.77 | 0.72 |
| Central Memory | 44.09±23.69 | 57.13±31.94 | 27.09±23.37 | 0.25 |
| Effector Memory | 12.81±13.64 | 11.98±15.09 | 20.14±9.86 | 0.30 |
| Effector | 5.49±4.76 | 4.04±4.51 | 21.41±13.11 | 0.04* |
| 1 Month |  |  |  |  |
| Total CD8+ | 52.78±16.75 | 53.91±15.75 | 50.29±13.07 | 0.91 |
| Naïve | 32.74±20.39 | 37.02±36.84 | 43.61±33.14 | 0.93 |
| Central Memory | 42.89±23.57 | 48.68±38.11 | 38.92±24.38 | 0.79 |
| Effector Memory | 12.66±9.81 | 7.51±5.78 | 10.26±11.76 | 0.46 |
| Effector | 11.71±13.42 | 6.79±11.84 | 7.21±3.39 | 0.49 |
| 3 Months |  |  |  |  |
| Total CD8+ | 49.29±7.87 | 47.11±15.21 | 45.36±13.06 | 0.94 |
| Naïve | 37.92±34.41 | 42.10±36.49 | 45.24±34.32 | 0.87 |
| Central Memory | 44.43±27.24 | 41.62±39.48 | 32.99±22.06 | 0.87 |
| Effector Memory | 12.91±13.22 | 6.09±7.72 | 12.81±14.58 | 0.42 |
| Effector | 4.83±4.53 | 10.19±11.00 | 8.97±2.49 | 0.43 |
| 6 Months |  |  |  |  |
| Total CD8+ | 51.34±10.35 | 45.57±22.07 | 65.51±2.84 | 0.29 |
| Naïve | 28.46±25.72 | 12.97±6.83 | 23.17±25.86 | 0.59 |
| Central Memory | 53.87±26.84 | 46.42±25.98 | 28.96±20.96 | 0.41 |
| Effector Memory | 10.57±8.81 | 29.05±21.11 | 25.87±26.31 | 0.20 |
| Effector | 7.08±12.44 | 11.55±11.21 | 22.01±21.41 | 0.13 |
| 12 Months |  |  |  |  |
| Total CD8+ | 40.29±13.23 | 36.52±10.14 | 52.29±21.19 | 0.50 |
| Naïve | 26.03±25.68 | 35.39±34.99 | 32.08±33.09 | 0.82 |
| Central Memory | 53.50±21.66 | 44.73±24.83 | 26.04±1.19 | 0.19 |
| Effector Memory | 16.37±14.74 | 16.04±11.42 | 27.39±28.12 | 0.80 |
| Effector | 4.10±4.19 | 3.86±1.64 | 14.49±3.76 | 0.01* |
